# *Pediococcus acidilactici* pA1c^®^ Improves the Beneficial Effects of Metformin Treatment in Type 2 Diabetes by Controlling Glycaemia and Modulating Intestinal Microbiota

**DOI:** 10.3390/pharmaceutics15041203

**Published:** 2023-04-10

**Authors:** Miriam Cabello-Olmo, María Oneca, Raquel Urtasun, María J. Pajares, Saioa Goñi, José I. Riezu-Boj, Fermín I. Milagro, Josune Ayo, Ignacio J. Encio, Miguel Barajas, Miriam Araña

**Affiliations:** 1 Biochemistry Area, Department of Health Science, Public University of Navarre, 31008 Pamplona, Spain; 2 Genbioma Aplicaciones S.L. Polígono Industrial Noain-Esquíroz, Calle S, Nave 4, 31191 Esquíroz, Spain; 3 IDISNA Navarra’s Health Research Institute, 31008 Pamplona, Spain; 4 Center for Nutrition Research, Department of Nutrition, Food Science and Physiology, University of Navarra, 31008 Pamplona, Spain; 5 Centro de Investigación Biomédica en Red de la Fisiopatología de la Obesidad y Nutrición (CIBERobn), Instituto de Salud Carlos III, 28029 Madrid, Spain

**Keywords:** *Pediococcus acidilactici*, probiotic, metformin, high-fat diet, diabetes, glycaemia, intestinal microbiota

## Abstract

Type 2 diabetes (T2D) is a complex metabolic disease, which involves maintained hyperglycemia, mainly due to the development of an insulin resistance process. Metformin administration is the most prescribed treatment for diabetic patients. In a previously published study, we demonstrated that *Pediococcus acidilactici* pA1c^®^ (pA1c) protects from insulin resistance and body weight gain in HFD-induced diabetic mice. The present work aimed to evaluate the possible beneficial impact of a 16-week administration of pA1c, metformin, or the combination of pA1c and metformin in a T2D HFD-induced mice model. We found that the simultaneous administration of both products attenuated hyperglycemia, increased high-intensity insulin-positive areas in the pancreas and HOMA-β, decreased HOMA-IR and also provided more beneficial effects than metformin treatment (regarding HOMA-IR, serum C-peptide level, liver steatosis or hepatic *Fasn* expression), and pA1c treatment (regarding body weight or hepatic *G6pase* expression). The three treatments had a significant impact on fecal microbiota and led to differential composition of commensal bacterial populations. In conclusion, our findings suggest that *P. acidilactici* pA1c^®^ administration improved metformin beneficial effects as a T2D treatment, and it would be a valuable therapeutic strategy to treat T2D.

## 1. Introduction

Type 2 diabetes mellitus (T2D) is a chronic metabolic disease, mainly characterized by an increased blood glucose concentration produced by deficient insulin secretion by pancreatic islet β cells in the context of impaired insulin sensitivity [1,2]. Type 2 diabetes accounts for more than 90% of patients with diabetes, and the incidence and prevalence of the disease continue to rise globally, becoming one of the leading causes of death worldwide [3]. Moreover, although T2D is mainly linked to adults, lately, the prevalence of this disease has dramatically increased in adolescents and young adults. Genetic predisposition partially explains the individual susceptibility to developing T2D, but an unhealthy diet and a sedentary lifestyle are determinant factors of the current global epidemic [4].

Metformin is one of the most commonly prescribed treatments for T2D because of its outstanding ability to decrease plasma glucose levels [5]. Metformin is a biguanide drug able to suppress glucose production in the liver through gluconeogenesis inhibition, trigger peripheral glucose utilization in the liver, intestine and skeletal muscle and increase insulin sensitivity [6]. Recently, changes derived from metformin administration and gut microbiota have been reported [7]. Some authors found an increase in the *Akkermansia* spp. population derived by metformin treatment, which is related to glucose homeostasis in high-fat diet (HFD)-induced obese mice [8,9]. Another reported target of metformin action is the shift towards short-chain fatty acids (SCFAs) producing bacteria mostly in the *Firmicutes* and *Proteobacteria* phyla [10,11]. Finally, some actions on bile acids mediated by microbiota have been described, observing increased bile acid synthesis and a reduced circulating LDL cholesterol concentration [12]. However, although several possible pathophysiological mechanisms have been suggested to explain these beneficial clinical effects of metformin, its precise mechanism of action remains to be elucidated.

In the last few years, evidence has accumulated to support the link between deranged glycemia and intestinal dysbiosis, on the basis that microbiota-derived compounds are implicated in many physiological functions [13]. In short, the mechanisms of action of probiotics in metabolic diseases include changes in energy metabolism, immunomodulation, and alterations in the mucosal barrier and gut microbial composition [14].

In a previously published study, we demonstrated the antidiabetic effects of *Pediococcus acidilactici* pA1c in a murine model of HFD-induced T2D. The obtained results indicated that pA1c improved HFD-induced T2D-derived insulin resistance (IR) and intestinal histology, as well as protecting mice from body weight (BW) increases [15].

Since metformin has become the usual treatment for T2D patients, we wanted to elucidate whether the combination of pA1c and metformin may cause deleterious outcomes such as hypoglycemia or, on the contrary, the combination of pA1c and metformin could improve the described antidiabetic effects for pA1c.

Therefore, we investigated the protective effect of metformin and pA1c combination in a T2D HFD-induced mice model. The findings of this study may provide new insight for the pharmacological approach to T2D in the future.

## 2. Materials and Methods

### 2.1. Experimental Design

Forty male C57BL/6 mice (Charles River Laboratories) aged 11 weeks were acclimated for 1 week with a standard chow diet. At this point, the animals were randomly divided and allocated into four groups (*n* = 10 each): (1) Control group (Co group), animals receiving an HFD; (2) metformin group (Met group), animals receiving HFD plus metformin; (3) pA1c group (pA1c group), animals receiving HFD plus a probiotic formulation with pA1c; and (4) animals receiving HFD plus a combination of metformin and a probiotic formulation with pA1c (Met + pA1c group).

The high-fat diet (TD.06414, Envigo, Tekla, Indianapolis, IN, USA) contains 60% of kcal from fats (detailed information in Supplementary Table S1). The probiotic pA1c, which has been evaluated in a previous study [15], has a patent application regarding “Probiotics for regulating blood glucose [PCT/EP2020/087284; WO2021/123355A1]” and is registered in the Spanish Collection of Type Cultures (CECT) (Reference CECT 9879). The strain is regarded as safe according to the Qualified Presumption of Safety (QPS) by the European Food Safety Authority (EFSA).

During the study, all groups had ad libitum access to food and water. To verify food acceptance, food and drink intake were visually confirmed by one investigator throughout the experimental study. In addition, food intake was monitored every 2 weeks.

At the end of the study (17 weeks), all animals were sacrificed by cervical dislocation for the collection of blood after 12 h (overnight) fasting and tissue samples (liver, pancreas and small intestine). Blood samples were centrifuged for 8 min at 2000 rpm, and serum samples were stored at −80 °C until analysis. Tissue samples for quantitative real-time PCR were cut and frozen at −80 °C immediately after collection. Tissue samples for histological analysis were washed with PBS and fixed in a formaldehyde solution (10%) for 24 h. Stool samples were collected at the end of the study for analysis of the fecal microbiota composition using a metagenomics approach.

**Figure 1:**
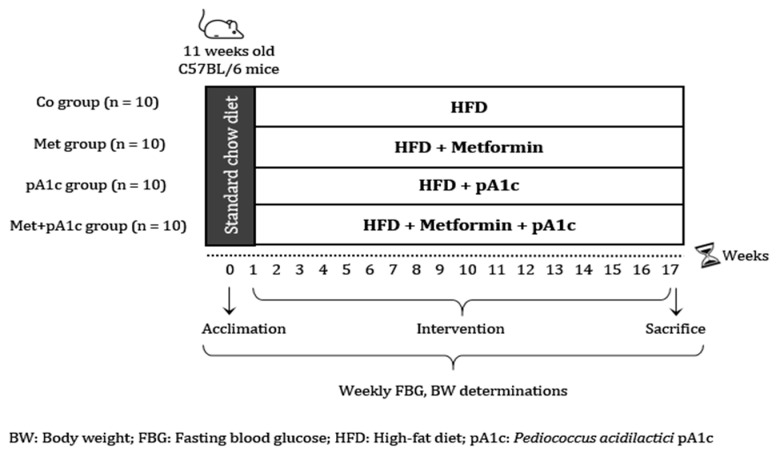
Experimental design.

A summary of the experimental design is presented in Figure 1. All animal procedures were performed under protocols approved by the Institutional Committee on Care and Use of Laboratory Animals (CEEA, University of Navarra) (Protocol number: CEEA/017-20).

### 2.2. Diets and Metformin Preparation

The probiotic was administered within the diet, while metformin was administered in the drinking water. Both mixtures were prepared weekly in our laboratory under sterile conditions and kept at 4 °C until use.

For the preparation of the diet (pA1c and Met + pA1c groups), commercial HFD was enriched with a probiotic formulation containing pA1c. The dose of the probiotic in the diet was adjusted to 1 × 10^10^ CFU per day/animal, and the microbiological load in the diet was confirmed using the classic plate count method (37 °C and 5% CO_2_ during 48 h). Before the study, pA1c survival in HFD was determined to confirm that animals received viable microorganisms during the intervention.

Water with metformin was prepared (Met and Met + pA1c groups), dissolving the appropriate amount of metformin in sterile water (0.3 g/kg of body weight) [8,16,17]. The metformin was in the form of metformin hydrochloride and was a kind gift from Ricardo Molina S.A.U (Barcelona, Spain). The concentration of metformin in the water was adjusted weekly to the average body weight in each group. Before the study, we confirmed the correct metformin solubility in water for the used concentration.

### 2.3. Body Weight, Fasting Blood Glucose and Intraperitoneal Glucose Tolerance Test

Body weight (BW) was recorded once weekly. Further, 12 h (overnight) FBG was determined once a week using a glucometer (Accu-chek Aviva, Roche, Basel, Switzerland), and 12 h fasting blood samples were obtained from the tip of the tail vein for the analysis of biochemical parameters. In addition, an intraperitoneal glucose tolerance test (IPGTT) was performed at 14 wks. For that, 12 h fasted animals received glucose (Baxter, Valencia, Spain) intraperitoneally (1.5 g/kg of body weight), and glycemia was determined as described for FBG at different time points (baseline, 20, 40, 60, 90 and 120 min) after glucose injection.

### 2.4. Biochemical Analysis

Serum biochemical analyses were determined at the end of the study. Serum ALT and AST transaminases, HDL cholesterol, LDL cholesterol, total cholesterol (TC), non-esterified fatty acids (NEFAs) and triglyceride (TG) levels were determined using a Cobas c-311 (Roche Diagnostics, Basel, Switzerland) analyzer.

Serum GLP-1 and leptin concentrations were measured by enzymatic methods using commercial kits (Abyntek Biopharma, Derio, Spain). We also determined C peptide (Abyntek Biopharma, Derio, Spain) as an indirect estimation of insulin synthesis in the pancreas.

The homeostasis model assessment of IR (HOMA-IR) and β-cell function (HOMA-β) was estimated using the following formulas: HOMA-IR = serum C peptide (ng mL^−1^) × blood glucose (mmol L^−1^)/22.5; HOMA-β = 20 × serum C peptide (ng mL^−1^)/(blood glucose (mmol L^−1^) − 0.35).

### 2.5. Tissue Collection and Histological Analysis

Fixed tissue samples (pancreas, liver and small intestine) were embedded in paraffin and cut into 3 µm-thick sections. Sections were stained with hematoxylin and eosin (H&E) for light microscopy examination, and in the small intestine, an additional section was stained with periodic acid–Schiff (PAS). Detailed information of the protocol used for the preparation of the tissue samples and the immunolabeling is described in [15].

All the slides were digitized using a histology slide scanner Aperio CS, running under the Scan Scope Console software (v.10.2.0.2352, Leica Biosystems, Inc. 1700 Leider Lane, Buffalo Grove, IL, USA), and further analyzed using Fiji software.

In the pancreas sections, we evaluated insulin-positive areas and scored pancreatic islets following a histological intensity score, as described in [15]. We analyzed five photographs (10×) from six different sections for each animal (*n* = 8 for each group).

In small intestine sections, we measured the percentage of GLP-1-positive cells per total cells in five random fields (20×) in five different sections (*n* = 8 for each group). In PAS-stained small intestine sections, we determined the percentage of goblet cells per total enterocytes in ten random areas per animal (*n* = 8 for each group).

In H&E-stained liver sections, we analyzed steatosis (expressed as a percentage of the area occupied by lipid droplets) in eight random images (20×) for each animal (*n* = 8 for each group).

### 2.6. Quantitative Real-Time PCR (RT-qPCR)

Frozen (−80 °C) liver tissues were used for total RNA extraction using RNeasy Mini Kit (Qiagen, Germantown, MD, USA) according to the manufacturer’s instructions. The purity and concentration were determined via spectrophotometry using Nanodrop One (Thermo Scientific, Madrid, Spain). Then, cDNA synthesis (RT) was performed using SuperscriptTM IV VILOTMRT Premix with enzDNaseTM (Invitrogen, Carlsbad, CA, USA) following the manufacturer’s instructions. The mRNA expression levels of hepatic glycogenesis-related genes (glucose-6-phosphatase (*G6Pase*), phosphoenolpyruvate carboxykinase (*Pepck*) and glucokinase (*Gck*)), lipid metabolism-related genes (*Srebp*, *Fasn*, *Pparα*, *Pparγ*) and inflammation-related genes (interleukine-1β (*Il-1β*) and interleukine-6 (*Il-6*)) were analyzed and further normalized using (*Rplp0*) as a house-keeping gene. Quantitative real-time PCRs (qPCR) were performed with IQ SYBR Green Supermix (Bio-Rad, Hercules, CA, USA) in a CFX Connect TM Real-time system (Bio-Rad, Hercules, CA, USA). Data are expressed as the relative mRNA normalized to *Rplpo* and analyzed according to the comparative cycle threshold method (2^−∆∆CT^). Primer sequences for the targeted mouse genes and sources are shown in Supplementary Table S2.

### 2.7. Fecal Metagenomic Analysis

At the end of the study, fecal samples (*n* = 5 per group) were collected and immediately frozen at −80 °C for metagenomics analysis. Fecal DNA was extracted from the stool samples and sequenced on the MiSeq platform (Illumina, San Diego, CA, USA) in CIMA Labs Diagnostics (Pamplona, Spain). Bacterial DNA isolation was carried out with Promega-Maxwell^®^ RSC equipment using the Maxwell RSC Fecal Microbiome DNA Kit (Promega Corporation, Madison, WI, USA). For each DNA sample, the V3-V4 hypervariable regions of the 16S rRNA gene were amplified using specific primers (Illumina, San Diego, CA, USA). The 16S rRNA sequences were filtered using BaseSpace™ Sequence Hub (Illumina), and the Amplicon Sequence Variant (ASV) abundance matrix was generated. Finally, taxonomy was assigned [18] using Ribosomal Database Project (RDP) classifier (v3 May 2018 DADa2 32bp).

### 2.8. Statistical Analysis

Data are presented as mean ± standard deviation (SD). All statistical procedures were performed using GraphPad Prism 8.0.1 software. Venn diagrams were made using the venny 2.1.0 software “https://bioinfogp.cnb.csic.es/tools/venny/ (accessed on 1 February 2023)”. Statistical significance was set at *p* < 0.05. * denotes *p* < 0.05, ** denotes *p* < 0.01 and *** denotes *p* < 0.001.

Statistical differences in microbiota abundances between groups were tested at species, genus and family levels using filtered data with a minimum count of 4 counts in at least 20% of samples and a variance higher than 10% of the inter-quantile range. Data were transformed to Centered Log-Ratio (CLR) to avoid the problems from compositional data and analyzed using *t*-test/ANOVA methods.

Data were analyzed by using one-way analysis of variance (ANOVA) followed by Tukey’s post hoc test or the Kruskal–Wallis test followed by Dunn’s test for multiple comparisons, as appropriate. Metagenomic data were analyzed using MicrobiomeAnalyst platform [19]. Alpha diversity at feature level was analyzed with unfiltered data and using parametric test. Beta diversity was performed using the ordination-based method Principal Coordinate Analysis (PCoA), Bray–Curtis distance, statistical method PERMANOVA and data (feature level) normalized by relative log expression.

## 3. Results

### 3.1. Metformin, pA1c and Their Combination Attenuated BW Gain in HFD-Fed Mice

**Figure 2:**
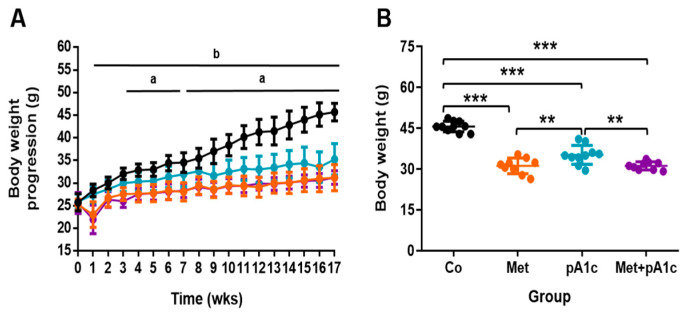
(**A**) Body weight progression (mean ± SD, n = 10 mice per group) and (**B**) scatter dot plot representing BW at the end of the study. ** *p* < 0.01; *** *p* < 0.001.; ^a^ significant differences Co vs. pA1c group; ^b^ significant differences Co vs. Met group and Co vs. Met + pA1c.

When we analyzed the impact of metformin and pA1c in BW, we observed significant differences between groups from wk 1 until the end of the study. Figure 2A shows that Co group presented a steep rise in BW, while BW progression in the other groups was steadier. We found differences between the Co group and pA1c group from wk 3 to 17, except for wk 7. In addition, we found differences between the Co group and both Met and Met + pA1c groups from wk 1 until wk 17. The two groups with metformin (Met and Met + pA1c groups) experienced a sharp decrease in BW values at wk 1, which coincides with the beginning of the treatment with metformin.

**Figure 3:**
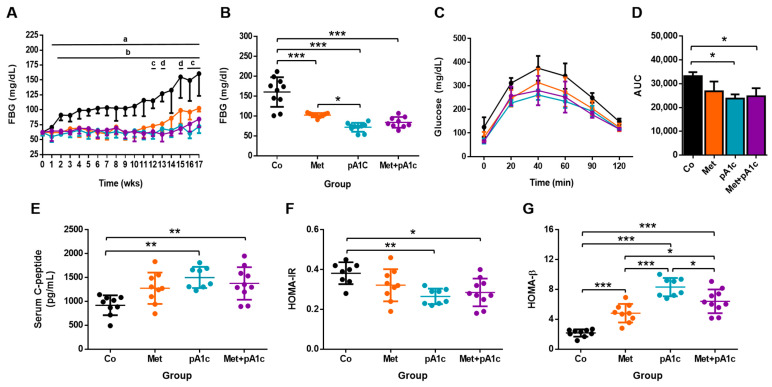
(**A**) FBG progression throughout the study (mean ± SD, *n* = 10 mice per group). Scatter dot plots representing (**B**) FBG at the end of the study; (**C**) IPGTT at week 14; (**D**) total AUC glucose at week 14; (**E**) serum C peptide, (**F**) HOMA-IR and (**G**) HOMA-β at the end of the study (*n* = 9–10 mice per group). * *p* < 0.05; ** *p* < 0.01; *** *p* < 0.001; ^a^ significant differences Co vs. pA1c group; ^b^ significant differences Co vs. Met group and Co vs. Met + pA1c; ^c^ significant differences Met vs. pA1c group; ^d^ significant differences Met vs. Met + pA1c.

From wk 1, food intake was recorded for each group every 2 wks (Supplementary Figure S1). What stands out is that food intake peaked in wk 3 in the two groups with metformin (Met group and Met + pA1c group), just after a drop in BW in these groups in wk 1 (Figure 3A). After that, however, such an effect disappeared, suggesting that animals became acclimatized to metformin, and food consumption fell to values closer to those in the Co and pA1c groups. The analysis indicated that food intake was comparable in all the study groups.

No statistically significant differences were found in the BW at the beginning of the study between groups (average BW 25.6 g, range 21.5–28.5 g). At the end of the study, however, we found great differences between groups (*p* < 0.001). As shown in Figure 2B, the Co group weighed more than the other groups (45.7 ± 1.9 g; *p* < 0.001 vs. all the groups), and the pA1c group (35.2 ± 3.5 g) weighed significantly more than the two groups with metformin (Met group: 31.2 ± 2.9 g; Met + pA1c group: 31.2 ± 1.5 g; *p* < 0.01 vs. both Met and Met + pA1c groups).

We estimated the average BW gain in each group as change (%) from baseline BW. Data showed that animals in the Co group exhibited the greatest BW gain (78.2 ± 14.6%), which was significantly greater than the rest of the groups (Met group: 30.6 ± 15.9%, *p* < 0.001 vs. Co group; pA1c group: 36.5 ± 14.9, *p* < 0.05 vs. Co group; Met + pA1c group: 22.8 ± 14.2%, *p* < 0.001 vs. Co group). The Met group, pA1c group and Met + pA1c group presented comparable values.

### 3.2. pA1c and Its Combination with Metformin Mitigated Glucose Dysregulation, Attenuated Insulin Resistance and Preserved Pancreatic β-Cell Functioning Better Than Metformin

FBG values in the Co group appeared to be separated from the other groups from wk 2 (Figure 3A) and maintained greater FBG values as well as greater dispersion throughout the study. We found significant differences between the Co and pA1c groups (from wk 1 to wk 17), between Co and both Met and Met + pA1c groups (from wk 2 to wk 17), between Met and pA1c groups (at wk 12, 16, and 17) and, finally, between Met and Met + pA1c groups (at wk 13 and 15). In parallel, from wk 10 onwards, the Met group had growing FBG values, suggesting that this treatment became less effective over time.

All study groups had comparable FBG at the beginning of the study (average FBG 62.3 ± 4.4 mg/dL, range 55.0–71.0 mg/dL). Nevertheless, at the end of the study, we observed great differences between groups (Figure 3B). We found a similar tendency to that in BW, where the Co group had significantly higher FBG compared with the rest of the study groups (160.6 ± 37.2 mg/dL; *p* < 0.001 vs. all the groups). On top of that, we found significant differences between the Met and pA1c groups (Met group: 102.6 ± 5.4 mg/dL, pA1c group: 72.0 ± 11.0 mg/dL; *p* < 0.05). In the case of FBG, however, the Met + pA1c group (84.3 ± 13.4 mg/dL) had comparable values to those in the Met group and pA1c group (Figure 3B). On the other hand, the area under the curve (AUC) of the IPGTT at 14 wks reflects significant differences in glucose tolerance between pA1c-administered groups and the control group (Figure 3C,D).

It is apparent from Figure 3E that interventions with the probiotic led to changes in the metabolism of insulin since both pA1c and Met + pA1c groups presented significantly greater serum levels of C peptide at the end of the study. Metformin alone, however, did not change C-peptide concentrations at the end of the study (17 wks).

Figure 3F shows that HOMA-IR scores were slightly impacted by the treatments, especially by the probiotic supplementation. Both the pA1c and Met + pA1c groups presented significantly lower HOMA-IR values as compared to the Co group (*p* < 0.01 and *p* < 0.05 in pA1c and Met + pA1c groups, respectively). HOMA-β, however, was dramatically influenced by all the treatments, and at the end of the study, HOMA-β scores were significantly different between all the groups, especially with the Co group (Figure 3G). The lowest value corresponded to the Co group (2.2 ± 0.5) and the highest to pA1c group (8.3 ± 1.2), whose mean value was about 3.6-times the value in control animals.

**Figure 4:**
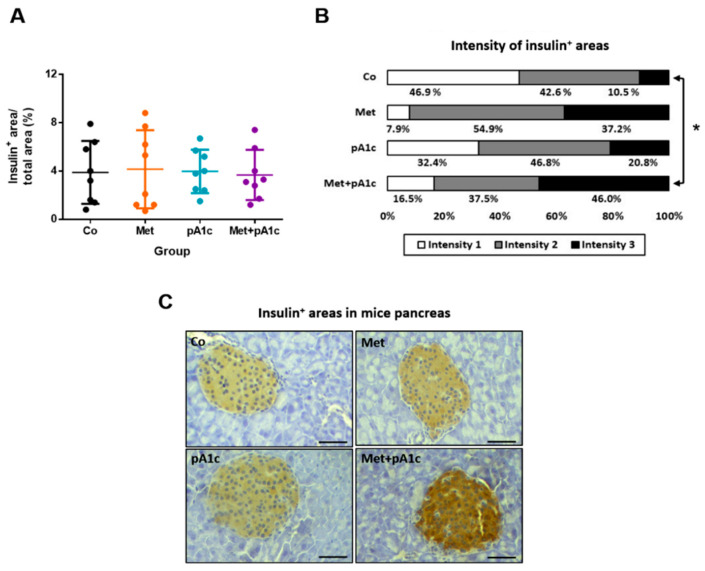
(**A**) Scatter dot plot representing the percentage of insulin^+^ areas and (**B**) intensity of insulin^+^ areas in each study group (*n* = 8 mice per group). (**C**) Representative images of pancreas sections immunostained for insulin in Co, Met, pA1c and Met + pA1c groups (scale bars, 50 µm). * *p* < 0.05.

We estimated the ratio of insulin-positive areas and total pancreas area, and we did not identify significant differences between groups (Figure 4A). However, when we quantified the intensity of insulin^+^ areas (intensity values: 1, 2 or 3), we found significant differences (Figure 4B). The percentage of intensity 1 and 2 areas was comparable in all the study groups. However, regarding the percentage of intensity 3 areas, the Met + pA1c group had the highest proportion and significantly exceeded the percentage in the Co group (*p* < 0.05). Representative images are shown in Figure 4C.

### 3.3. All the Treatments Protected Mice from High Leptin Levels and Provoked Marked Changes in Lipid Profile

**Figure 5:**
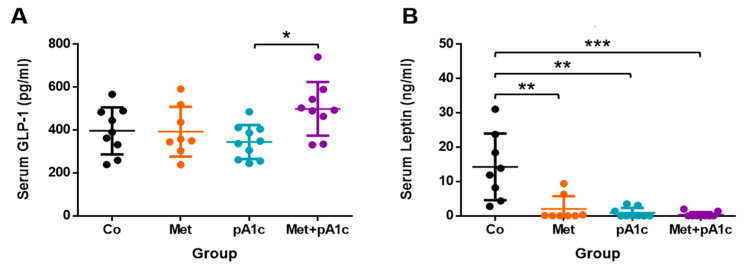
Scatter dot plots representing serum (**A**) GLP-1 and (**B**) leptin levels in the experimental groups (*n* = 8–9 mice per group). * *p* < 0.05; ** *p* < 0.01; *** *p* < 0.001.

Data indicated a wide range of serum GLP-1 concentration, with values ranging from 244.1 to 590.9 pg/mL. The pA1c and Met + pA1c groups had the lowest and highest mean values, respectively (396.1 ± 109.7 and 498.8 ± 125.0 pg/mL). GLP-1 levels were significantly different between both groups (*p* < 0.05) (Figure 5A). Additionally, we also appreciated dissimilar tendencies between the Co and Met + pA1c groups; however, the variability in the samples within groups did not allow us to identify significant differences.

Remarkably, data from serum leptin indicated that treatments with metformin, probiotics and their combination had a profound effect on this adipokine. As shown in Figure 5B, the Co group reached very high values (17.8 ± 13.9 ng/mL), which overwhelmingly surpassed values in the other groups (2.0 ± 3.7, 0.9 ± 1.4, and 0.4 ± 0.8 ng/mL in Met, pA1c and Met + pA1c groups, respectively), showing significant differences between groups (*p <* 0.01 vs. Met and pA1c groups, *p* < 0.001 vs. Met + pA1c group).

Regarding other biochemical parameters, we found certain differences between study groups (Table 1). Starting with transaminases, mice in the pA1c and Met + pA1c groups had lower ALT values as compared to the Co group (*p* < 0.01 vs. pA1c group and *p* < 0.05 vs. Met + pA1c group). By contrast, no remarkable differences were found in AST values.

On the other hand, we found that the interventions impacted some lipid profile markers. TC and HDL were reduced by the three treatments compared with the Co group (TC: *p* < 0.0001 vs. Met group, *p* < 0.01 vs. pA1c and Met + pA1c groups; HDL: *p* < 0.05 vs. pA1c group; *p* < 0.01 vs. Met and Met + pA1c groups). In the case of LDL, only Met and Met + pA1c treatments were able to significantly reduce serum levels (*p* < 0.001 vs. Met group, *p* < 0.001 vs. Met + pA1c group).

The intervention had a profound effect on serum TG, which significantly varied between groups. In a similar manner to TC and HDL, Met and Met + pA1c groups presented lower TG concentrations than the Co group (*p* < 0.001). However, the pA1c group had significantly greater TG levels than the Co group (*p* < 0.001). It is the single most striking observation to emerge from the data, since TG values in the pA1c group were exceptionally greater as compared to the Met and Met + pA1c groups (*p* < 0.01 in both groups).

Regarding NEFAs, only animals in the Met + pA1c group had reduced levels compared with controls (*p* < 0.05). Similar to what we found in TG, the pA1c group had the highest NEFA concentration and was significantly different from both Met and Met + pA1c groups (*p* < 0.01 in both groups).

### 3.4. Metformin, pA1c and Their Combination Had a Mild Impact on Genes Involved in Energy Metabolism and Inflammation

**Figure 6:**
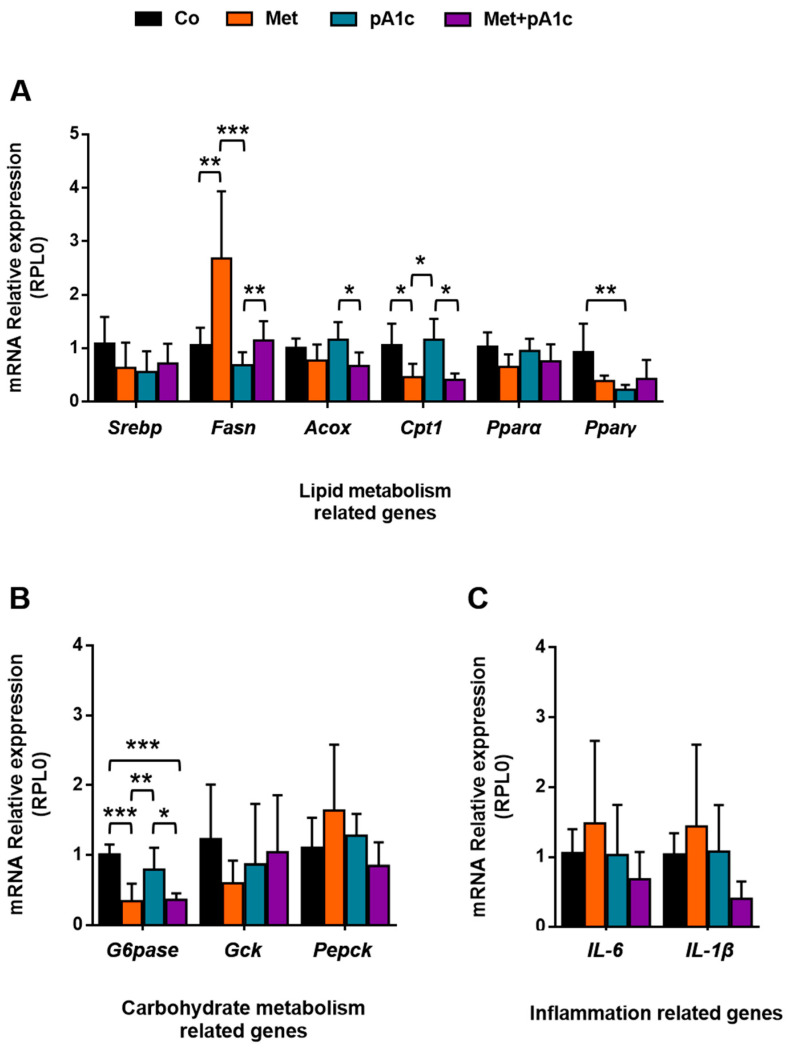
Hepatic mRNA relative expression of genes related to (**A**) lipid metabolism, (**B**) carbohydrate metabolism and (**C**) inflammation. *Acox:* Acetyl-CoA oxidase*; Cpt1:* carnitine palmitoyltransferase 1; *Fasn*: fatty acid synthase; *Gck*: glucokinase; *G6pase*: glucose-6-phosphatase; *Il-6*: interleukin 6; *Il-1β*: interleukin 1β; *Pepck*: phosphoenolpyruvate carboxykinase; *Pparα*: peroxisome proliferator-activated receptor alpha; *Ppaγ*: peroxisome proliferator-activated receptor gamma; *Srebp*: sterol regulatory element-binding protein. All data expressed as mean ± SD (*n* = 6 mice per group). * *p* < 0.05; ** *p* < 0.01; *** *p* < 0.001.

Regarding gene expression in liver-metabolism-related genes (Figure 6A), we found significant differences in four of the six analyzed genes. The treatment with metformin upregulated the mRNA expression of *Fasn*, which is involved in lipogenesis. Its relative expression was significantly greater in the Met group compared to the other groups (*p* < 0.01 vs. Co and Met + pA1c groups; *p* < 0.001 vs. pA1c group). *Cpt1* was also altered, and we identified significant differences between Co and Met, Met and pA1c and pA1c and Met + pA1c (*p* < 0.05 in all comparisons). By contrast, we only found significant differences in *Acox* between pA1c and Met + pA1c (*p* < 0.05). Concerning *Pparγ*, we identified statistical differences between the Co and pA1c groups (*p* < 0.05). None of the treatments altered *Pparα* or *Srebp* expression in the liver.

We also examined three genes related to carbohydrate metabolism (Figure 6B). The G6Pase enzyme was downregulated in both Met and Met + pA1c groups (*p* < 0.001), while the pA1c group preserved values comparable to the Co group and significantly differed with respect to both groups (*p* < 0.01 and *p* < 0.05 vs. Met and Met + pA1c groups, respectively). In relation to *Pepck* and *Gck*, we did not find significant differences between groups. The impact of metformin alone deserves attention, since the Met group displayed a tendency for greater and lower values in *Pepck* and *Gck*, respectively, and behaved differently than the other study groups.

With respect to inflammatory markers in the liver (Figure 6C), we found a tendency for greater values in the Met group; however, there were no significant differences between groups.

### 3.5. pA1c and Its Combination with Metformin Had a Protective Effect against Liver Steatosis in HFD-Fed Mice

**Figure 7:**
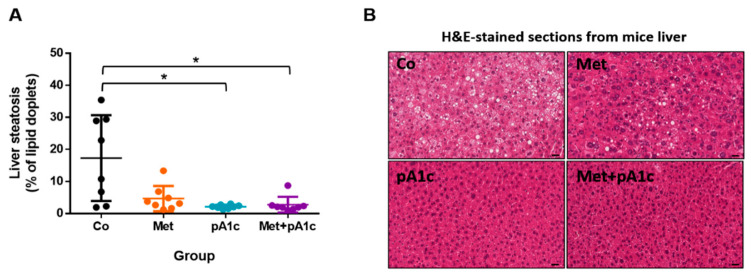
(**A**) Impact of the treatments on hepatic lipid accumulation expressed as percentage of lipid droplets in histological sections (*n* = 8 mice per group). (**B**) Representative images of H&E-stained hepatic sections (scale bars, 25 µm). * *p* < 0.05.

As shown in Figure 7A, the analysis of liver steatosis showed a significant decrease in the percentage of steatosis in the pA1c and Met + pA1c groups in comparison to the Co group (*p* < 0.05), suggesting a protective effect against liver damage. In Figure 7B, representative images can be viewed.

### 3.6. Metformin and Its Combination with pA1c Sustained Mucus-Secreting Goblet Cell Proliferation but Did Not Impact on the Release of GLP-1 in the Intestine

**Figure 8:**
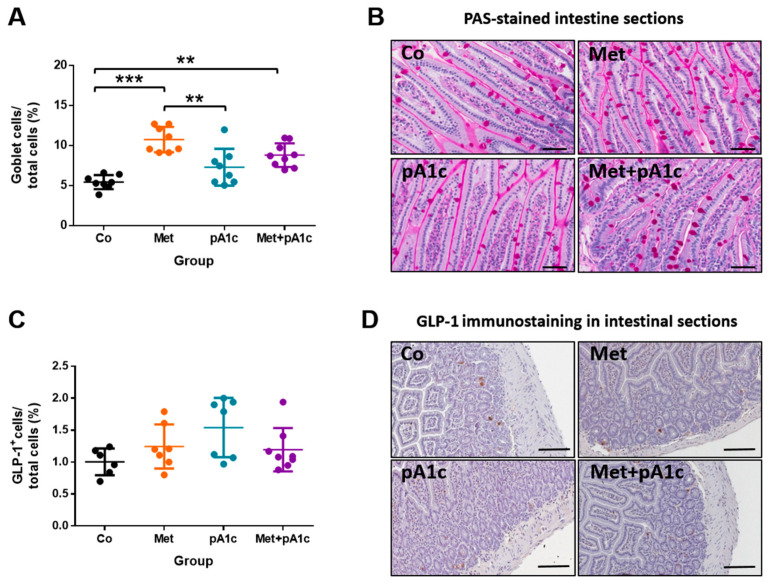
(**A**) Scatter dot plots representing the percentage of goblet cells in intestinal tissue and (**B**) representative images of PAS-stained intestinal sections (*n* = 8 mice per group) (scale bars, 50 µm). (**C**) Scatter dot plots representing the percentage of GLP-1^+^ cells and (**D**) representative images of immunostaining for GLP-1 in intestinal sections (*n* = 8 mice per group) (scale bars, 100 µm). ** *p* < 0.01; *** *p* < 0.001.

Small intestine analysis revealed a significant increase in the percentage of goblet cells with the two metformin treatments (Co vs. Met group: *p* < 0.001; Co vs. Met + pA1c group: *p* < 0.01). In addition, the percentage in the Met group was significantly greater than that in the pA1c group (*p* < 0.01) (Figure 8A). Representative images are shown in Figure 8B. On the other hand, the treatments did not influence the production of GLP-1 in intestinal cells, and there were no significant differences in the percentage of GLP-1^+^ cells between groups (Figure 8C). Representative images are shown in Figure 8D.

### 3.7. Metformin, pA1c and Their Combination Modulated Intestinal Microbiota Composition

In total, 19 samples were included in the metagenomic analysis because one outlier was detected in the pA1c group (*n* = 5 in Co, Met and Met + pA1c groups and *n* = 4 in pA1c group).

**Figure 9:**
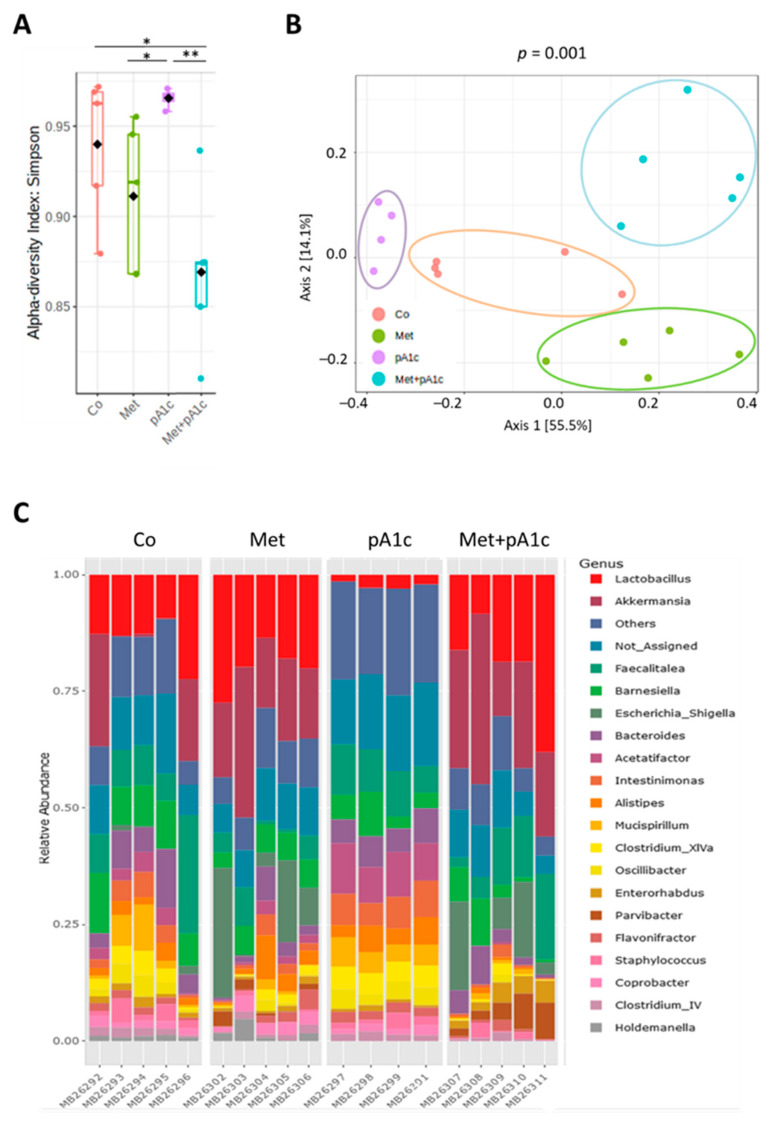
Modulation of gut microbiota diversity. (**A**) Alpha-diversity determined at species level by Simpson index. (**B**) Species beta-diversity indices performed using the ordination method based on Principal Coordinate Analysis (PCoA) and Bray–Curtis distance assessed using PERMANOVA test. (**C**) Relative abundance of the 20 most abundant genera (*n* = 4–5 mice per group). * *p* < 0.05; ** *p* < 0.01.

The α-diversity of gut microbiota, determined at the species level via Simpson index, significantly differed between groups (*p* < 0.01). Interestingly, alpha-diversity was significantly lower in the Met + pA1c group as compared to the Co group (*p* < 0.03). In addition, the groups that received metformin alone (Met group) or in combination with pA1c (Met + pA1c group) displayed significantly lower α-diversity compared with single probiotic treatment (pA1c group) (*p* < 0.02 and *p* < 0.009, respectively) (Figure 9A). We also analyzed the β-diversity represented by Principal Coordinate Analysis (PCoA) and Bray–Curtis distance of the species profiles. The statistical analysis showed significant differences among the four groups (*p* < 0.001 determined using PERMANOVA). As seen in Figure 9B, the pA1c group presented the highest similarities between group samples, while samples in the other three groups were more heterogeneous. When analyzed by genus, the most striking changes observed were that *Lactobacillus* and *Akkermansia* are almost absent in the pA1c group. In contrast, metformin administration had the opposite effect in these two genera, showing greater relative abundance in both genera when compared with the pA1c group and being close to the Co group (Figure 9C).

**Figure 10:**
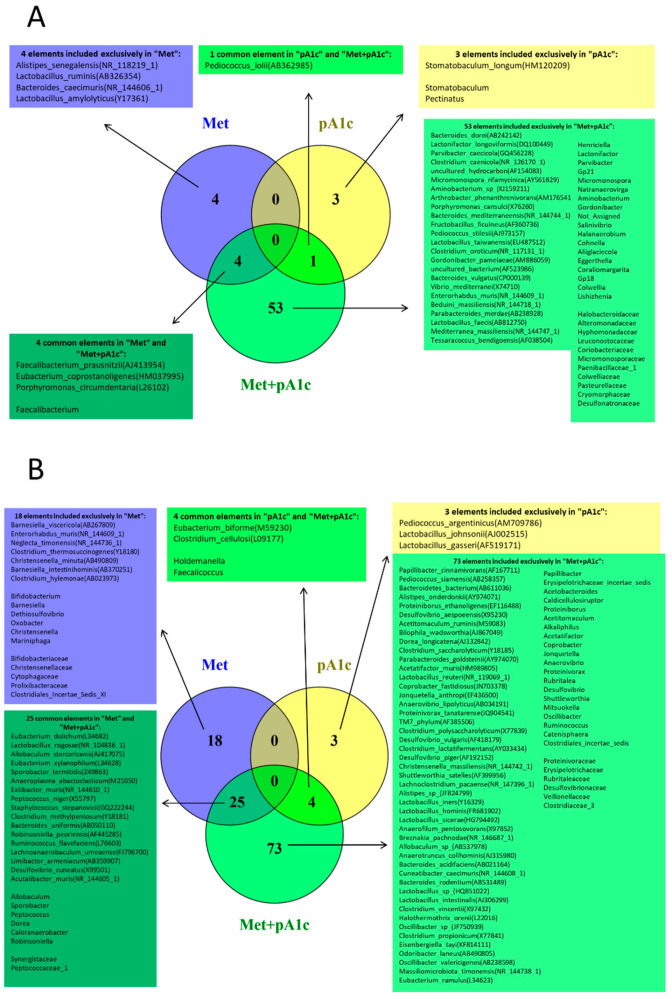
Venn diagrams of the identified taxa showing (**A**) the upregulated and (**B**) downregulated taxa in the Met, pA1c and Met + pA1c groups compared with the Co group (*n* = 4–5 mice per group).

In order to analyze the taxa (at the species, genus and family levels) that presented significant differences in abundance, Student’s t tests were performed between the control group and each of the treated groups (Co vs. Met, Co vs. pA1c and Co vs. Met + pA1c). Figure 10 shows the results of each test as Venn diagrams to compare the taxa that are upregulated (Figure 10A) or downregulated (Figure 10B). The results showed that the only common upregulated taxon after pA1c treatment (pA1c and Met + pA1c groups) was the species *Pediococcus lolii.* We found a total of four elements upregulated in the metformin-treated groups (Met and Met + pA1c), including the Gram-positive taxa *Faecalibacterium*, *Faecalibacterium praustnizii* and *Eubacterium coprostanoligenes* and the Gram-negative species *Porphyromonas circumdentaria*. The treatment that provoked the most profound changes in fecal microbiota was Met + pA1c, which significantly upregulated 53 microbiota components, including 11 families, 18 genera and 24 species. Additionally, some elements were only upregulated with single probiotic or metformin treatment. Three taxa were exclusively increased in the pA1c group (*Stomatobaculum longum*, *Stomatobaculum* and *Pectinatus*), and four species were augmented in the Met group (*Alistipes senegalensis*, *Bacteroides caecimuris*, *Lactobacillus ruminis* and *Lactobacillus amylolyticus*) (Figure 10A).

On the other hand, the Venn diagram of downregulated taxa among the three groups compared with the Co group indicates that four elements were present in both the pA1c and Met + pA1c groups (*Eubacterium biforme, Clostridium cellulosi, Holdemanella* and *Faecalicoccus)* and 25 elements were common in Met and Met + pA1c groups, including 2 families, 6 genera and 17 species. In addition, 3 elements were present exclusively in the pA1c group (*Pediococcus argentinicus, Lactobacillus johnsonii* and *Lactobacillus gasseri),* and 18 elements were present exclusively in the Met group (5 families, 6 genera and 7 species). Again, the largest number of downregulated microbiota components was found within the Met + pA1c group, with 73 elements, including 6 families, 20 genera and 47 species (Figure 10B). No common upregulated/downregulated elements were found between the three groups nor between the Met and pA1c groups. Plots representing the relative abundance of the cited taxa are available in Supplementary Figure S2.

Since the objective of the study was to examine the effect of metformin and pA1c administration, particular attention was given to the presence of *Pediococcus* strains in the studied groups. We found a high presence of *Pediococcus lolii* in those groups that received the probiotics (pA1c and Met + pA1c groups), this taxon being very scarce in the Co group and almost absent in the Met group (Supplementary Figure S3). Interestingly, this pattern was not found with any other *Pediococcus* strain and it should be noted that *P. lolii* has been described as a *P. acidilactici* strain because of the high sequence similarity between them [20,21].

## 4. Discussion

A series of experimental studies have documented the benefits of the biological properties of different *Pediococcus* strains, including resistance to digestion [22], good probiotic attributes, such as aggregation, coaggregation and adhesion properties [23,24], as well as antagonism against pathogenic microorganisms [25]. We recently demonstrated that *Pediococcus acidilactici* pA1c counteracts the effects of high-glucose exposure in *C. elegans* through the insulin signaling pathway [26] and its antidiabetic effect in T2D HFD-induced mice [15]. In the present research, we took a step further and evaluated the effects of the probiotic combination with metformin, which is considered a first-line pharmacological treatment against T2D [6], to test whether this combination would offer advantages compared to the current treatment. The results of this study showed that the administration of pA1c, metformin and their combination had a normoglycaemic effect, reduced IR and strengthened pancreatic function in HFD-fed mice. It should be noted that the best results were observed with the combination of pA1c and metformin. Nevertheless, we observed that each treatment had a different impact on mice’s physiology and gene expression, and it appears that metformin and pA1c could potentially exert their beneficial effects through different mechanisms of action. These points will be discussed in more detail in the following paragraphs.

As mentioned above, the three treatments (pA1c, metformin and the combination of both) remarkably reduced glycaemia during the study but, apparently, metformin alone was not able to sustain low FBG values in the long term, and it lost its ability to attenuate blood glucose at the end of the study. These results are in contrast with previous results from T2D murine models, where metformin administration reduced FBG efficiently after 6 weeks [27], 13 weeks [28] or 6 months [16]. The FBG observations are in line with results from C peptide and HOMA-IR, where the intake of pA1c (alone or combined with metformin) showed clear advantages over the intake of metformin alone. Animals in these two groups demonstrated better β-cell functioning, suggesting that pA1c exerted insulinotropic effects in our model. In addition, the absence of extremely low FBG values in all the study groups is a positive outcome and suggests that both the probiotic alone and its combination with metformin are safe, and they do not present a hypoglycemic risk for diabetic patients.

Notably, the histological analysis insinuated that the combination of pA1c and metformin had a more substantial impact on pancreas β-cell islets, since animals under this treatment showed greater immunohistochemical reactivity to insulin, indicating higher insulin production, probably due to protection of the beta-cell mass against glucotoxicity exerted by poor glycemic control. Considering this, the simultaneous use of pA1c and metformin would be of special interest for diabetic subjects with poor endocrine pancreatic function. Regarding the GLP-1 analysis, it is well known that GLP-1 can control β-cell mass proliferation and activity, and it is a key factor for the treatment of T2D [29]. Our previous preclinical study [15] and other animal experiments using probiotics in models of T2D and HFD-fed mice [16,30,31] showed a significant increase in serum GLP-1. Some authors have discussed that metformin can increase GLP-1 levels, either directly, by enhancing their release, or indirectly, by reducing dipeptidyl peptidase-4 (DPP-4) activity [32], a protease that has become an important pharmacological target for T2D because of its ability to regulate increasing cleavage and, consequently, control glucose levels [33]. In subjects with T2D [34] and HFD-fed mice [16,35], metformin administration increased GLP-1 levels. On the contrary, in the present research, no significant differences in GLP-1 levels were found in either blood or intestinal tissue, not even in the metformin-treated group.

It has previously been demonstrated that the treatment of T2D patients with metformin exerts a glucose-lowering effect, mostly by inhibiting hepatic gluconeogenesis [6,13,36]. We observed that the treatments with metformin and its combination with pA1c had a meaningful effect on hepatic *G6Pase,* but not in *Pepck* or *Gck*. Nevertheless, the impact of metformin in hepatic *G6Pase* and *Gck* relative expression is still controversial, and we can find animal studies reporting increased [17] or decreased [16] expression of *G6Pase* and *Gck* following metformin treatment.

Next, a molecule that could help us to elucidate the role of the combination of metformin with the pA1c is leptin, an adipokine that can act in the hypothalamus and govern food intake [37]. In our study, the enormous differences in leptin levels between control and treatment groups did not translate into changes in food intake but did affect BW, which was found to be increased in control animals. These results match those of previous studies [16,30] but are contrary to those of Shin et al. [8], Yu et al. [38] and Lin et al. [39], who found no effect of metformin administration on BW compared to a non-treated group, and Zulkawi et al. [17], who reported a decline in food intake following metformin treatment. Even though we have no data on body composition, serum leptin results strongly suggest that the three treatments, and especially the combination of pA1c and metformin, led to fewer fat depots as compared to control animals. Although white adipocytes are specialized in fat storage, fat can also accumulate in non-adipose tissues, forming the so-called ectopic lipid deposition. In conditions of IR, the activation of hepatic de novo lipogenesis (DNL) can contribute to triglyceride droplet accumulation in the tissue, thus causing metabolic stress and contributing to the pathogenesis of non-alcoholic fatty liver disease (NAFLD) [40]. In this line, we found that the group with the highest HOMA-IR values (Co group) also presented the greatest level of *Srebp* as well as the highest hepatic steatosis. SREBP is an important transcription factor controlling DNL and the synthesis of TG [41,42], and with time, its activation can also contribute to hepatic IR [43]. Even though significant differences were not found, a tendency for greater hepatic *Srebp* mRNA levels in the control group could be observed, which is in good agreement with results from liver steatosis. Indeed, a previous animal study reported a significant correlation between *Srebp* and TG in the liver tissue [44]. Notwithstanding, in addition to DNL, there are other processes controlling hepatic fat accumulation, such as fatty acid oxidation and circulating lipid input and output [43], which makes it difficult for us to identify how our treatments impacted hepatic lipid metabolism and storage.

One of the reasons for hepatic lipid accumulation is fatty acid mobilization from adipose tissue [45]. Thus, we would expect to find more steatosis in those groups with the highest adipose tissue mass. Though body composition was not determined, data from BW and leptin revealed important differences between control animals and those treated with metformin, pA1c or their combination, and this could also indicate differences in fat deposition. Regarding liver tissue, metformin inhibited HFD-induced fatty liver disease in *ob/ob* mice [39] and decreased the incidence of fatty liver disease in clinical trials [46], while another investigation in subjects with T2D [47] found no change in hepatic fat content following metformin treatment.

While *Pparα* was unaltered by metformin, *Cpt1* was negatively affected and remained unchanged in the group treated with the probiotic alone. Further, *Acox*, another gene linked to fatty acid oxidation, seems to behave in a similar manner to *Cpt1*. All this may suggest that metformin impacted fatty acid catabolism [48].

Following on from the foregoing, in our study, we found that pA1c-treated groups presented lower hepatic steatosis and also lower *Fasn* expression that the two groups treated with metformin, even though *Srebp*, a major transcriptional regulator in DNL, was not significantly changed by any treatment [43]. In a previous experiment, *Fasn* was also found to be downregulated with pA1c in *C. elegans* [26]. In our study, *Pparγ* was also downregulated in the pA1c group. At the same time, the metformin group showed hepatic steatosis along with an abrupt rise in *Fasn*, which is a key rate-limiting enzyme involved in DNL and a marker of lipogenesis [37]. Instead, serum TG and NEFAS were lower in the two groups receiving metformin, and once again, better results were obtained when metformin and pA1c were co-administered. These findings might suggest that pA1c can counteract hepatic steatosis and attenuate DNL, which could be explained by an increased lipid output, while metformin may impact lipid metabolism, particularly by inhibiting fatty acid oxidation and activating DNL. The fact that the group receiving both metformin and pA1c had lower hepatic *Fasn* expression, a lower degree of steatosis and lower serum TG and NEFAS is good evidence of this.

According to ALT levels, pA1c-treated groups presented lower hepatotoxicity, and data from pro-inflammatory markers also support the hypothesis that the intake of the probiotic was helpful in attenuating liver inflammation induced by HFD administration. Interestingly, while previous research reported the anti-inflammatory properties of metformin [49,50], at the end of our study, the group receiving metformin alone tended for greater IL-6 and IL-1β, and its combination with pA1c successfully normalized cytokine expression. Considering that hepatic steatosis and damage are risk factors for further liver diseases [45], this outcome is of great relevance and suggests that pA1c intake could provide some protection against liver damage in diabetic patients, probably through metabolic pathways that occur in parallel to counteract the observed long-term deleterious actions of metformin.

It has already been reported that metformin beneficially enhanced the goblet cell population [8]. Goblet cells are specialized secretory cells that produce mucus and other molecules, and, along with antibacterial and defensive components released by other epithelial cells, constitute a physical–chemical barrier that maintains epithelial integrity and helps to regulate the entrance of potential pathogens and unknown substances [51,52]. Under an HFD, oxidative stress dysregulates mucus production and impairs its protective function [53], and inflammation can prompt lipopolysaccharide endotoxemia, which is involved in IR and T2D development [54]. In our study, metformin (alone and combined with pA1c) had a greater effect on intestinal histology than the probiotic treatment, leading to a higher amount of mucus-producing goblet cells, which is in agreement with previously published studies. For example, a study in a mice model of ulcerative colitis showed that metformin can improve intestinal health by reducing the inflammatory tone and enhancing mucus production, and such effects seemed to be partially mediated by the gut microbiota [55]. Similarly, another study performed on mice with AOM/DSS-induced colon cancer and streptozotocin-induced T2D mice concluded that metformin helped in reducing intestinal inflammation, preserving colon histology and decreasing tumor cell proliferation, and the authors suspected a microbiota-mediated effect as well [56]. The authors hypothesized that deranged microbiota could prevent the beneficial effects of metformin, and the incorporation of a probiotic would help favor metformin’s beneficial effects. In this respect, a previous study suggested that gastrointestinal symptoms with metformin use could be due to the enrichment in *Escherichia* species [57]; thus, microbiota-targeted interventions such as probiotics could help to recover a healthy microbiota and re-establish gut homeostasis. Indeed, a previous study confirmed that the combination of metformin and the probiotic *Bifidobacterium bifidum* G9-1 (BBG9-1) resulted in improved gastrointestinal symptoms (diarrhea and constipation scores) in subjects with T2D [58]. Given all the above, and in agreement with the results reported in this study, it seems that the simultaneous administration of probiotic strains and metformin would provide more benefits than the single treatments.

We know from previous research that intestinal microbiota are a relevant risk factor, mediating disease progression and severity, and that all forms of diabetes *mellitus* are accompanied by significant microbiota alterations [59,60]. In the same line, a solid body of literature has shown that metformin can provoke profound changes in the gut microbiota composition up to strain levels [13] and that metformin’s effectivity is partially due to microbial mediation [10,61,62]. We performed a deep analysis of the microbiota composition in the four experimental groups, intending to identify microbiota patterns linked to metformin, pA1c or their combination, and, subsequently, understanding the potential mechanism which motivated the physiological effects observed in our study. It has been reported that metformin decreased microbiota α-diversity in HFD-fed mice [63], while another study in the same animal model found no significant effect on α-diversity [35], and an open-label clinical trial found a relevant enrichment in α-diversity [64]. Such heterogeneity between studies denotes a lack of consensus on the effect of metformin on microbiota diversity. In our study, metformin was not as efficient as the probiotic treatment in improving microbiota diversity in HFD-fed mice since the supplementation with metformin alone or combined with pA1c led to the lowest overall bacteria diversity, while both control and pA1c groups had higher and comparable values of α-diversity. Results from a previous report following gut microbiota transfer indicate that greater microbiota diversity is associated with improved IR [10], and a previous case–control study also concluded that α-diversity was negatively linked to parameters indicating T2D severity, suggesting better metabolic control with high microbiota diversity [65]. In our research, single probiotic treatment and control group presented the highest α-diversity, which does not match the studies above.

Next, we found that *Pediococcus lolii* was the most abundant *Pediococcus* species in the microbiota samples, particularly in the pA1c groups. We know from previous publications that some *P. lolii* strains and *P. acidilactici* clustered together with high sequence similarity [20,21], and this suggests that the counts of *P. lolii* correspond to the counts of our probiotic. This would confirm that pA1c presents good probiotic properties, leading to its survival and gut colonization. In addition, the fact that its combination with metformin also led to high counts in the microbiota suggests that this specific probiotic strain has good tolerance to the antidiabetic drug, and they could be administered together. Whether *Pediococcus* spp. abundance and/or the observed effects in this study last through the time when probiotic intake is interrupted is yet to be confirmed.

The treatment with pA1c (alone or combined with metformin) was related to a downregulation of three taxa that have been found enriched in different pathological conditions. One of them, *Eubacterium biforme*, was found enriched in *Helicobacter pylori* infections in children [66] and also in adults with metabolic-dysfunction-associated fatty liver disease (MAFLD) [67,68]. Further, *Holdemanella* was significantly greater in subjects with liver fibrosis [69], while *Faecalicoccus* was more abundant in patients with Chron’s disease, ulcerative colitis, multiple sclerosis and rheumatoid arthritis [70].

Regarding the relative abundance found in the study, the rise in *Akkermansia* with metformin (alone or combined with pA1c) is in good agreement with previous reports in HFD-fed mice [8], *db/db* mice [71] and subjects with T2D [10]. This outcome is very interesting for T2D management since *Akkermansia* is a well-known beneficial bacterium widely studied for its favorable effect on intestinal integrity [72] and is often depleted in T2D [61,73] and also in earlier stages such as prediabetes [65]. In a similar way to *Akkermansia, Lactobacillus* was considerably reduced in the pA1c group. The information is unclear regarding *Lactobacillus spp*., and although this genus was found enriched in diabetic patients, previous findings pointed out a species effect; thus, it would be more appropriate to check individual *Lactobacillus* species and not total *Lactobacillus* abundance [74,75].

In addition, metformin failed to markedly affect microbiota diversity; it substantially influenced its structure and led to selective modulations of specific gut microbial phylotypes. Alone or co-administered with the probiotic, metformin significantly upregulated *Faecalibacterium*, *Faecalibacterium prausnitzii* and *Eubacterium coprostanoligenes*, which are butyrate-producing bacteria (BPB), and *Porphyromonas circumdentaria*, which also produced acetic and butyric acid in vitro [76]. Along with *Roseburia* spp. and *Eubacterium rectale, F. praustnizii* is a major BPB in the human microbiota [77]. This commensal bacterium is associated with better metabolic health [78] and was found depleted in prediabetes, type 1 and type 2 diabetic subjects [61,78], while it was more abundant in individuals with normal glucose tolerance [79]. In gnotobiotic mice, it was associated with increased mucus production and goblet cells and, therefore, a stronger intestinal barrier [80], in agreement with the increase in goblet cells found in our study after metformin treatments. To continue, *E. coprostanoligenes* was significantly increased in HFD-fed hyperlipidemic rats treated with a strain of *P. acidilactici* isolated from traditional fermented brewing, and the ability to attenuate dyslipidemia by metabolizing cholesterol was attributed to the bacterium [81]. In a mice model of periodontitis, *E. coprostanoligenes* negatively correlated with serum HbA1c [82], and this bacterium was also linked to lower FBG and body weight in another experiment in HFD-fed mice [83].

Among the 25 common downregulated taxa with metformin treatment, special attention was given to the SCFA-producing *Allobaculum*. In line with our findings, this genus was also found to be significantly enriched in HFD-fed mice and was also positively correlated to fasting induction factor (FIAF), which is a key mediator in lipid metabolism [84]. Contrary to our findings, previous works on Goto-Kakizaki rats [85] and HFD-fed rats [86] found that both metformin and berberine increased *Allobaculum* abundance, and the same effect was found with other antidiabetic drugs [87] and probiotic treatments [31].

One of the goals of this research was to assess the suitability of the simultaneous administration of metformin and pA1c, and the microbiota analysis confirmed that the combined treatment provoked different changes from those observed with metformin or pA1c separately. Among the long list of taxa altered in the Met + pA1c group, the upregulation of *Parvibacter* and *Coriobacteriaceae* was interesting. The first was found enriched in HFD-fed mice following treatment with prebiotics, which improved insulin sensibility and protected them from hepatic steatosis and dyslipidemia [88], and a similar outcome was found in *ob/ob* mice given inulin [89]. In addition, this bacterium was negatively related to low-density lipoprotein–cholesterol levels [90]. Concerning *Coriobacteriaceae*, a recent report found that this bacterial family and others were markedly enriched in subjects with low inflammatory scores [91] and in healthy subjects, as compared to women with gestational diabetes [92]. Another publication attributed most of the beneficial effects of Roux-en-Y gastric bypass in T2D patients to this bacterial family [93].

With regard to taxa downregulated in the Met + pA1c group, we would like to emphasize two specific taxa: *Acetatifactor muris* and *Bilophila wadsworthia*. *Acetatifactor muris* is present in the intestine of obese mice [94] and it was found upregulated in T2D patients treated with metformin [95]. *Bilophila wadsworthia* produces a toxic gas that provokes intestinal damage [96] and was linked to systemic inflammation in specific pathogen-free mice [97].

Lastly, we found it interesting that *Enterorhabdus muris* was downregulated with metformin while it was upregulated with the Met + pA1c treatment. Even though our results disagree with previous research where metformin significantly increased *Enterorhabdus* abundance in HFD-fed mice [63], they are in line with a previous study in which xylooligosaccharide administration normalized the abundance of this genus, which was abnormally enriched in individuals with prediabetes [98].

### Limitations, Strengths and Future Studies

Other studies carried out on probiotics and the combination with metformin have been described in the literature, both in murine models [28,99] and in humans [58,100,101] in the context of T2D. In the same line, other studies tested this combination in different clinical contexts, such as ulcerative colitis [55], colorectal cancer [56], non-alcoholic steatohepatitis [102] and polycystic ovarian syndrome [103]. However, this was the first study to check the compatibility between one antidiabetic drug, metformin, and the probiotic strain pA1c. Despite all these promising data, we are aware that some potential limitations need to be considered. We acknowledge that the administration of metformin in the drinking water does not allow one to control the real dose ingested by each mouse and has limitations, and there is a possibility that dissimilar results would have arisen if we had given it via oral gavage, like other studies [8,28]. Further, we believe that the limited sample size prevented us from finding significant differences in some of the studied parameters.

Future studies should concentrate on different doses of metformin to find the lowest dose that retains beneficial effects, thus reducing the risk for adverse events thanks to the combination with pA1c. According to a previous study [104], the dose of metformin is of great importance and can dramatically determine its beneficial or deleterious effects; thus, the amount of metformin should be carefully defined in future studies. On top of that, more experiments are needed to elucidate the pharmacokinetics behind pA1c, and further studies are required to determine the effects of long-term exposure to the probiotic and also its combination with metformin.

## 5. Conclusions

To our knowledge, this is the first study combining metformin and a *Pediococcus acidilactici* strain, reporting that the combined treatment helped alleviate hyperglycemia. It appears that the beneficial effects of metformin and pA1c are independent; however, given the absence of a sharp fall in glycemia, we consider that there may be some communication or interplay between both mechanisms, which prevents hypoglycemia. We believe that pA1c contributes to a better body composition by controlling the adipose tissue and prevents metabolic imbalances by impacting intestinal physiology and commensal microbiota. Thus, more experiments are needed to clarify this issue.

Our findings are congruent with multiple lines of evidence indicating the efficacy of probiotic microorganisms to prevent or attenuate T2D, and they support the hypothesis that pA1c is a suitable antidiabetic treatment, which could be combined with metformin to improve the management of T2D and, eventually, reduce the required dose of the metformin treatment.

## Figures and Tables

**Table 1:** Biochemical parameters after the treatments.

Parameters	Co Group	Met Group	pA1c Group	Met + pA1c Group
ALT (U/L)	79.3 ± 58.7	38.5 ± 30.5	29.1 ± 11.9 **	26.5 ± 6.0 *
AST (U/L)	124.4 ± 48.6	94.4 ± 51.9	111.7 ± 33.2	113.0 ± 32.6
TC (mg/dL)	223.7 ± 48.9	141.4 ± 39.0 ***	167.2 ± 17.5 **	156.6 ± 26.0 **
HDL (mmol/L)	4.3 ± 0.7	3.1 ± 0.8 **	3.4 ± 0.6 *	3.1 ± 0.5 **
LDL (mmol/L)	0.8 ± 0.3	0.2 ± 0.1 ***	0.4 ± 0.1	0.2 ± 0.1 ***
TG (mg/dL)	154.7 ± 29.6	90.9 ± 14.4 ***^###^	207.9 ± 30.3 ***	98.3 ± 13.7 ***^###^
NEFAs (mmol/L)	2.2 ± 0.2	1.8 ± 0.4 ^##^	2.4 ± 0.3	1.8 ± 0.4 *^##^

Abbreviations: ALT alanine aminotransferase; AST aspartate aminotransferase; HDL high-density lipoprotein; LDL low-density lipoprotein; TC total cholesterol; NEFAs non-esterified fatty acids; TG triglycerides; TC total cholesterol. Values represented as mean ± SD (*n* = 9–10 mice per group). * *p* < 0.05, ** *p* < 0.01, *** *p* < 0.001 vs. Co group. ^##^ *p* < 0.01, ^###^ *p* < 0.001 vs. pA1c group.

## Data Availability

Public database used in this study included Ribosomal Database Project (RDP; http://rdp.cme.msu.edu) and NCBI (http://www.ncbi.nlm.nih.gov/taxonomy) accessed on 1 February 2023. The datasets generated or analyzed during the current study are available on request from the corresponding author.

## Supplementary Materials

The following supporting information can be downloaded at: https://www.mdpi.com/article/10.3390/pharmaceutics15041203/s1, Table S1. Nutritional information TD.06414 (Envigo); Table S2. Sequences of primers used in gene expression analysis; Figure S1. Average food intake in all the experimental groups during the study; Figure S2. Abundance of (A) upregulated and (B) downregulated taxa; Figure S3. Abundance (nº of reads) of *Pediococcus lolii* in the study groups. References [105,106,107,108] are cited in the supplementary materials.
