# Supplementary material for: Pediococcus acidilactici pA1c® Improves the Beneficial Effects of Metformin Treatment in Type 2 Diabetes by Controlling Glycaemia and Modulating Intestinal Microbiota
Source: Pharmaceutics. 2023 Apr 10;15(4):1203. doi: 10.3390/pharmaceutics15041203 (PMC10143274; doi:10.3390/pharmaceutics15041203)
Supplement: Supplementary file 1 [file pharmaceutics-15-01203-s001.zip › pharmaceutics-2259041-SI.pdf]

Supplementary materials

Table S1. Nutritional information TD.06414 (Envigo)

Formula	g/Kg	Composition	% by weight	% kcal from
Lard	310.0	Fat	34.3	60.3
Casein	265.0	Carbohydrate	27.3	21.4
Maltodextrin	160.0	Protein	23.5	18.3
Sucrose	90.0	Kcal/g	5.1	
Cellulose	65.5			
Mineral Mix, AIN-93G-MX (94046)	48.0			
Soybean Oil	30.0			
Vitamin Mix, AIN-93-VX (94047)	21.0			
L-Cystine	4.0			
Calcium Phosphate, dibasic	3.4			
Choline Bitartrate	3.0			
Blue Food Color	0.1			

Information obtained from Envigo Teklad Diets

Table S2. Sequences of primers used in gene expression analysis.

Target gene	Forward/ Reverse	Primer sequence (5´ to 3´)	Source
Acox	F	CTATGGGATCAGCCAGAAAG	[105]
	R	AGTCAAAGGCATCCACCAA	
Cpt1	F	CGAGGATTCTCTGGAACTGC	[105]
	R	GGTCGCTTCTTCAAGGTCTG	
Fasn	F	AGCCATGGAGGAGGTGGTGAT	[106]
	R	GTGTGCCTGCTTGGGGTGGAC	
Gck	F	CTTCACCTTCTCCTTCCCTGTAA	[107]
	R	AAAGTCCCCTCTCCTCTTGATAG	
G6Pase	F	CACCGACTACTACAGCAACAGC	[107]
	R	AGAATCCCAACCACAAGATGAC	
Il-1β	F	TCGCTCAGGGTCACAAGAAA	[106]
	R	CATCAGAGGCAAGGAGGAAAAC	
Il-6	F	ACAAGTCGGAGGCTTAATTACACAT	[106]
	R	TTGCCATTGCACAACTCTTTTC	
Pparα	F	ACTGGTAGTCTGCAAAACCAAA	[106]
	R	AGAGCCCCATCTGTCCTCTC	
Pparγ	F	GCTGTTATGGGTGAAACTCTG	[108]
	R	GAATAATAAGGTGGAGATGCAGG	
Pepck	F	AGTCATCATCACCCAAGAGC	[107]
	R	GGGATGACATACATGGTGC	
Rplp0	F	AACATCTCCCCCTTCTCCTT	[106]
	R	GAAGGCCTTGACCTTTTCAG	
Srepb	F	CACTTCATCAAGGCAGACTC	[106]
	R	CGGTAGCGCTTCTCAATGGC	

Acox: acyl-coenzyme A oxidase; *Cpt1*: carnitine palmitoyltransferase 1; *Fasn:* fatty acid synthase; *Gck*: glucokinase; *G6Pase*: glucose 6 phosphatase; *Il-1β*: interleukine-1β; *Il-6*: interleukin-6; *Pparα*: peroxisome proliferator-activated receptor *α; Pparγ*: peroxisome proliferator-activated receptor *γ; Pepck*: phosphoenolpyruvate carboxykinase; *Rplp0*: ribosomal protein P0; *Srebp*: sterol regulatory element-binding protein.

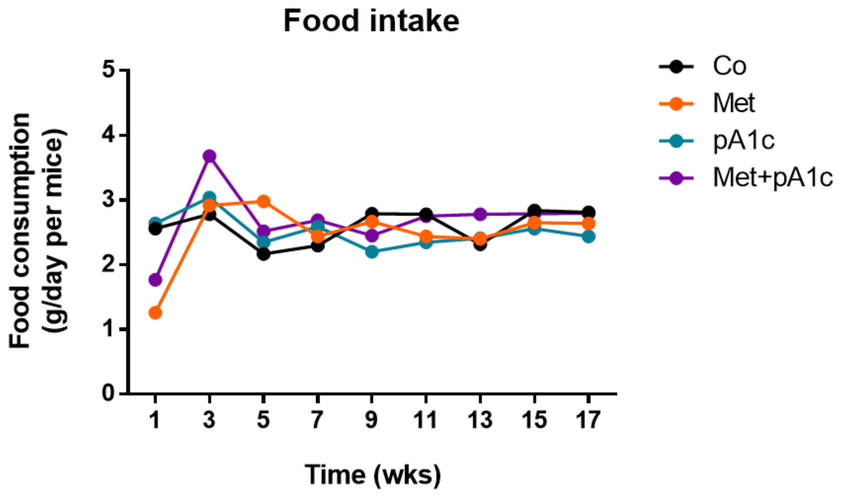

Figure S1. Average food intake in all the experimental groups during the study.

A

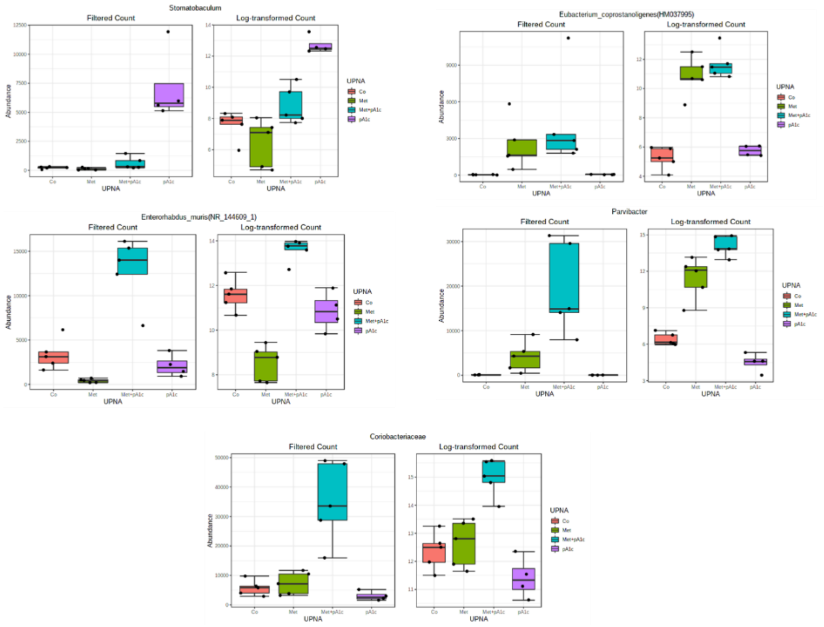

B

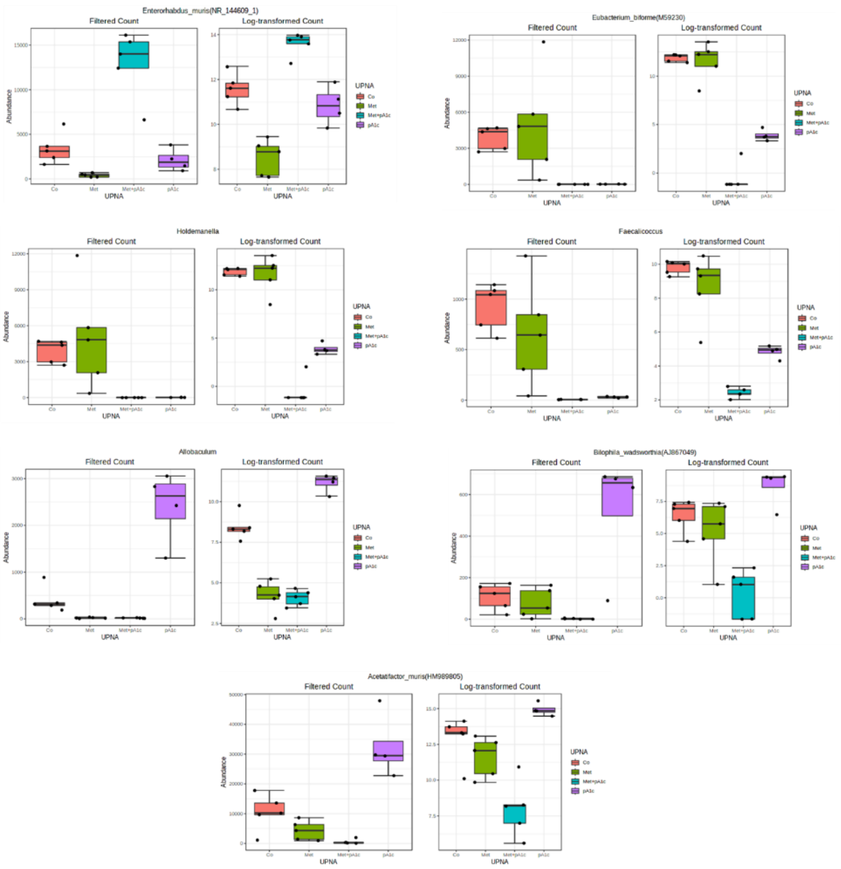

Figure S2. Abundance of (A) upregulated and (B) downregulated taxa.

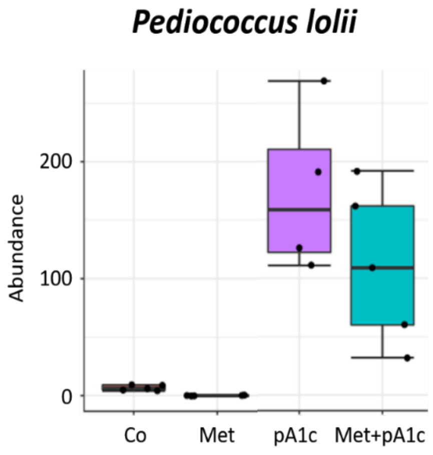

Figure S3. Abundance (nº of reads) of *Pediococcus lolii* in the study groups.
